# Association Between Low Testosterone and Perioperative Outcomes in Patients Undergoing Transurethral Prostate Surgery

**DOI:** 10.7759/cureus.74751

**Published:** 2024-11-29

**Authors:** Jasmine S Lin, Evan J Panken, Sai Kumar, Xinlei Mi, Edward Schaeffer, Robert E Brannigan, Joshua A Halpern, Daniel R Greenberg

**Affiliations:** 1 Urology, Cedars-Sinai Medical Center, Los Angeles, USA; 2 Urology, Northwestern University Feinberg School of Medicine, Chicago, USA; 3 Preventive Medicine, Northwestern University Feinberg School of Medicine, Chicago, USA

**Keywords:** benign prostatic hypertrophy, hypogonadism, laser photovaporization of prostate, low testosterone, sexual medicine, transurethral resection of prostate

## Abstract

Introduction

Low testosterone (T) is linked with frailty, which predicts poor postoperative recovery across many surgical procedures. Therefore, low T may impact perioperative outcomes for surgical patients. We sought to characterize the association between low T, frailty, and perioperative outcomes in patients undergoing transurethral resection of the prostate (TURP) and laser photovaporization of the prostate (PVP).

Materials and methods

We retrospectively reviewed men across our integrated healthcare system who underwent TURP or PVP with a recorded T level within one year prior to their procedure date. Low T was defined as a serum T <300 ng/dL. We compared clinical characteristics, lab values, and frailty, determined by the Hospital Frailty Risk Score (HFRS), of patients with low vs. normal T. Univariable and multivariable analyses were used to assess the association between low T and hospital readmission at 30, 90, and 180 days postoperatively.

Results

Among 175 patients who underwent either TURP or PVP, 86 (49.1%) had low T, and 89 (50.9%) had normal T. Patients with low T were older (68.7 ± 9.3 vs. 64.8 ± 11.8 years old, p = 0.016) and had longer postoperative length of stay (4.2 ± 10.5 vs. 1.4 ± 0.9 days, p = 0.03). Patients with low T had a significantly higher rate of readmission within 180 days (28% vs. 13%, p = 0.02). Low T was not independently associated with frailty. On univariable logistic regression, preoperative T was associated with readmission at 90 and 180 days. On multivariable regression, low preoperative T was no longer associated with 90-day readmission.

Conclusions

Almost half of the men undergoing transurethral surgery in our cohort had low T. Low T was independently associated with a higher risk of 180-day readmission on multivariable analysis. These findings indicate a possible prognostic role for low T screening in men undergoing transurethral prostatic surgery. Further studies are needed to determine whether preoperative treatment of low T can impact perioperative outcomes.

## Introduction

Low serum testosterone (T) is highly prevalent in the general population and increasingly so with age [1,2]. While low T is most commonly associated with symptoms of fatigue, erectile dysfunction, and decreased libido [3,4], it is also closely linked with anemia [5], decreased muscle mass [4,6], increased risk of cardiovascular disease, [7] and overall lower health-related quality of life [4]. Many of these signs and symptoms of low T overlap with those of frailty (e.g., decreased energy, muscle mass, and strength), and multiple studies have found a link between frailty and low T [8,9].

Frailty describes a decline in multiple physiological systems associated with aging, manifesting as a deterioration in the body’s ability to maintain homeostasis [10]. As a result, even minor physiologic stressors may exert disproportionately harmful effects on health over time. Common clinical presentations of frail patients include falls, delirium, and disability [10,11], but recent studies have shown frailty to be associated with poor postoperative recovery across a variety of surgical disciplines, including general surgery, spine surgery, cardiac surgery, and urologic surgery [12-15]. Given the relationship between low T and frailty, the presence of low T may adversely impact perioperative outcomes for surgical patients. However, there is an overall lack of scientific literature examining the association between low T and perioperative outcomes.

Considering the prevalence of low T in the general population, particularly older men [1,2], a large proportion of men undergoing transurethral procedures for benign prostatic hyperplasia (BPH) are likely to have low T. Therefore, we sought to characterize the prevalence of low T and the association between low T, frailty, and perioperative outcomes in patients undergoing transurethral resection of the prostate (TURP) and laser photovaporization of the prostate (PVP). We hypothesized that low T would adversely impact perioperative outcomes for patients undergoing these procedures. This article was previously presented as a meeting abstract at the 2023 American Urological Association Annual Meeting on April 30, 2023.

## Materials and methods

We performed a retrospective review of all men who underwent TURP or PVP by a urologist in our integrated healthcare system between 2002 and 2023. Inclusion criteria required patients to have a recorded T level within one year prior to their procedure date. These two procedures were identified using CPT codes 52601 and 52648, respectively. Men were considered to have low T if serum T was <300 ng/dL, in accordance with the American Urological Association Guideline on Testosterone Deficiency [16].

Frailty was assessed using the Hospital Frailty Risk Score (HFRS). The HFRS, developed and validated by Gilbert et al., employs a set of 109 predefined International Classification of Diseases, Tenth Revision (ICD-10) codes identified a priori as markers of frailty and uses them to calculate a score from 0 to 99 [17]. Patients are categorized based on these scores as low risk (score < 5), intermediate risk (score 5-15), or high risk (score > 15).

We compared clinical characteristics, lab values, and HFRS of patients with and without low T. Clinical characteristics included age at surgery, body mass index (BMI), race/ethnicity, and American Society of Anesthesiology (ASA) class. Descriptive statistics were calculated using a t-test for continuous variables and Fisher’s exact test or chi-squared test for categorical variables, as applicable.

Our primary outcome was the association between low T and hospital readmission at 30, 90, and 180 days postoperatively. Our secondary outcomes were the association between low T and mortality at 30, 90, and 180 days postoperatively. Univariable and multivariable logistic regression analysis was performed to assess primary and secondary outcomes using covariates, including age at surgery, BMI, frailty risk scores, and T. T was treated as a continuous variable. All tests of significance were two-sided, and a p-value <0.05 was considered statistically significant. Statistical analyses were performed in R (version 4.2.0, R Foundation for Statistical Computing, Vienna, Austria).

## Results

Among 175 patients who underwent either TURP or PVP, 86 (49.1%) had low T, and 89 (50.9%) had normal T. Mean preoperative T was 178.7 (± 99.3) ng/dL in the low T group vs. 470.7 (± 162.9) ng/dL in the normal T group (p < 0.001). Patients with low T were older (68.7 ± 9.3 vs. 64.8 ± 11.8 years old, p = 0.016) and had longer postoperative length of stay (4.2 ± 10.5 vs. 1.4 ± 0.9 days, p = 0.03), lower postoperative hemoglobin (12.5 ± 2.0 vs. 13.7 ± 1.7 g/dL, p < 0.001), and lower postoperative hematocrit (37.3% ± 6.1 vs. 40.9% ± 4.7, p < 0.001) (Table 1).

**Table 1:** Characteristics of men undergoing transurethral prostate surgery according to preoperative serum testosterone (normal ≥ 300 ng/dL) A p-value <0.05 was considered statistically significant ^*^Five men missing hemoglobin and hematocrit values ^^^Twelve men missing hemoglobin and hematocrit values ^1^Mean ± standard deviation; n (%) ^2^Welch two-sample t-test; Fisher’s exact test; Pearson’s chi-squared test HFRS, Hospital Frailty Risk Score; PVP, photovaporization of the prostate; TURP, transurethral resection of the prostate

	Low testosterone^1^ (n = 86)^*^	Normal testosterone^1^ (n = 89)^^^	p-value^2^
Age at surgery	68.7 ± 9.3	64.8 ± 11.8	0.016
BMI	28.5 ± 6.5	28.5 ± 5.3	>0.9
Procedure			0.9
TURP	78 (91%)	79 (89%)	-
PVP	8 (9.3%)	10 (11%)	-
Race			0.2
White	69 (80%)	73 (82%)	-
Asian	6 (7.0%)	1 (1.1%)	-
Black/African American	7 (8.1%)	9 (10%)	-
Declined/other	4 (4.7%)	6 (6.7%)	-
Ethnicity			0.06
Hispanic or Latino	1 (1.2%)	6 (6.7%)	-
Not Hispanic or Latino	76 (88%)	79 (89%)	-
Declined	9 (10%)	4 (4.5%)	-
ASA Class			0.13
0	35 (41%)	25 (28%)	-
1	3 (3.5%)	3 (3.4%)	-
2	24 (28%)	34 (38%)	-
3	21 (24%)	7 (30%)	-
4	3 (3.5%)	0 (0%)	-
HFRS	10.8 ± 12.2	10.5 ± 10.1	0.9
Low risk	35 (41%)	31 (35%)	0.7
Intermediate risk	29 (34%)	35 (39%)	-
High risk	22 (26%)	23 (26%)	-
Preop total testosterone (ng/dL)	178.7 ± 99.3	470.7 ± 162.9	<0.001
Total length of hospital stay (days)	4.17 ± 10.5	1.41 ± 0.93	0.031
Postop hemoglobin (ng/dL)	12.5 ± 2.1	13.7 ± 1.7	<0.001
Postop hematocrit (%)	37.3 ± 6.1	40.9 ± 4.7	<0.001
30-day readmission			0.7
No	77 (90%)	82 (92%)	-
Yes	9 (10%)	7 (8%)	-
90-day readmission			0.6
No	74 (86%)	80 (90%)	-
Yes	12 (14%)	9 (10%)	-
180-day readmission			0.020
No	61 (71%)	77 (87%)	-
Yes	25 (29%)	12 (13%)	-
30-day mortality			-
No	86 (100%)	89 (100%)	-
90-day mortality			-
No	86 (100%)	89 (100%)	-
180-day mortality			0.5
No	84 (98%)	89 (100%)	-
Yes	2 (2.3%)	0 (0%)	-

Of the 175 patients included in the analysis, 157 underwent TURP, while 18 underwent PVP. There were no significant differences between patients undergoing TURP vs. PVP in mean age at surgery (67.1 ± 10.6 vs. 63.2 ± 12.6 years old, p = 0.2), BMI (28.3 ± 6.0 vs. 30.3 ± 4.3, p = 0.09), HFRS (10.1 ± 9.7 vs. 15.5 ± 19.7, p = 0.3), or preoperative total T (322.0 ± 199.3 vs. 372.9 ± 197.5 ng/dL, p = 0.3). Compared to patients undergoing PVP, patients undergoing TURP were significantly more likely to have lower postoperative hemoglobin (12.9 ± 2.0 vs. 14.4 ± 1.1 g/dL, p < 0.001) and hematocrit (38.6% ± 5.8 vs. 43.5% ± 2.5, p < 0.001). There were no differences in readmission rates within 30 days (p = 0.3), 90 days (p = 0.2), or 180 days (p = 0.9) between patients undergoing TURP vs. PVP.

Patients with low T had a significantly higher rate of readmission within 180 days (29% vs. 13%, p = 0.019). However, there was no significant difference at 30 days (10% vs. 7.9%, p = 0.7) or 90 days (14% vs. 10%, p = 0.6) after surgery (Table 2). There were no deaths at 30 and 90 days in either group. There was no difference in mortality between the low and normal T groups at 180 days (Table 2).

**Table 2:** Univariable and multivariable logistic regression examining the association between postoperative readmission, testosterone, and frailty, as measured by the HFRS ^*^Association expressed as OR for univariable regressions and as adjusted OR for multivariable regressions. ^^^A p-value of <0.05 was considered statistically significant. HFRS, Hospital Frailty Risk Scores; OR, odds ratio; T, testosterone

	Univariable			Multivariable		
	OR^*^	95% CI	p-value^^^	Adjusted OR^*^	95% CI	p-value^^^
30-day readmission						
Preoperative total T	1.00	1.00, 1.00	0.2	1.00	1.00, 1.00	0.3
HFRS	1.00	0.95, 1.04	>0.9	1.00	0.94, 1.04	0.9
90-day readmission						
Preoperative total T	1.00	0.99, 1.00	0.035	1.00	1.00, 1.00	0.09
HFRS	1.02	0.98, 1.05	0.4	1.01	0.96, 1.04	0.7
180-day readmission						
Preoperative total T	1.00	0.99, 1.00	<0.001	1.00	0.99, 1.00	0.002
HFRS	1.04	1.01, 1.07	0.025	1.04	1.00, 1.07	0.07

In our cohort, low T was not independently associated with frailty (p = 0.9). On univariable logistic regression analysis, preop total T was associated with lower odds of 90-day readmission at 90 (OR 1.00, 95% CI 0.99-1.00, p = 0.035) and 180-day readmission (OR 1.00, 95% CI 0.99-1.00, p < 0.001) days. HFRS was also predictive of readmission at 180 days (OR 1.04, 95% CI 1.01-1.07, p = 0.025). BMI, race, and ASA class were not significantly associated with readmission at 30, 60, or 90 days on univariable regression analysis. On multivariable regression analysis, only low preoperative T was independently associated with 180-day readmission (OR 1.00, 95% CI 0.99-1.0, p = 0.002, respectively), whereas HFRS was no longer independently associated with 180-day readmission on multivariable analysis (OR 1.03, 95% CI 1.00-1.07, p = 0.067) (Table 2).

## Discussion

To our knowledge, this is the first study to examine the association between low T and perioperative outcomes for patients undergoing TURP and PVP. Our study found that patients with low T had a significantly longer postoperative length of stay, lower postoperative hemoglobin and hematocrit, and higher rates of readmission within 180 days. While low T was not independently associated with frailty, low T was independently associated with a higher risk of 180-day readmission.

Prior studies have investigated T as an independent predictor of outcomes following other types of urologic surgery. For example, several publications have found that men with low T have a significantly higher risk of artificial urinary sphincter (AUS) cuff erosion, which may be related to the specific local effects of low T on the tissue of the corpus spongiosum [18,19]. Treatment with T therapy was found to promote postoperative urethral angiogenesis, indicating a role for treatment to improve surgical outcomes. Likewise, low T at the time of kidney transplantation has been linked to death, reduced survival, and/or graft loss in some studies, while T therapy was found to increase graft survival [20,21].

The relationship between low T and BPH is complex [22]. T is trophic for prostatic tissue, and it is widely recognized that anti-androgenic therapies such as gonadotropin-releasing hormone (GnRH) agonists and 5-alpha reductase inhibitors can lead to decreased prostate volume [23,24]. Yet low T does not preclude the development of lower urinary tract symptoms due to BPH, as evidenced by the high number of men undergoing transurethral procedures with low T. Furthermore, the effect of low T on prostatic angiogenesis and fibrosis is not fully characterized, and this may have implications for intraoperative bleeding and operative approaches in men with low T.

Our results provide a signal that low T may be an independent risk factor for adverse perioperative outcomes after transurethral surgery, indicating a potential for T assessment and possibly treatment preoperatively. Future prospective studies are needed to better characterize the mechanism by which low T influences perioperative outcomes, as well as to determine whether there is a role for T replacement therapy preoperatively. Prior research has shown that T supplementation does not worsen lower urinary tract symptoms, including the results of the Testosterone Replacement Therapy for Assessment of Long-term Vascular Events and Efficacy Response in Hypogonadal Men (TRAVERSE) trial, the largest randomized trial of T replacement therapy to date [25,26]. Therefore, hypogonadism may be a modifiable risk factor before transurethral surgery for BPH.

While low T was not correlated with frailty in our study, frailty was an independent predictor of adverse perioperative outcomes. These findings expand upon recent studies that found an association between frailty, postoperative complications, and adverse lower urinary tract symptom outcomes in patients undergoing transurethral prostate procedures for BPH [27-29]. Taken together, these studies suggest a possible role for preoperative frailty assessment for risk stratification and frailty-reducing interventions.

Our findings should be interpreted within the context of several limitations. First, this is a retrospective study examining men who had a recorded T level within one year prior to their surgery date. There is a potential for selection bias as these men may have undergone T testing for various reasons, including signs or symptoms of T deficiency, exposure to risk factors for T deficiency, or fertility evaluation. This selection bias is also reflected in the high prevalence of low T in our cohort compared to other similarly aged cohorts from longitudinal aging studies [2]. Second, we defined low T based on a single laboratory test and not based on repeat measurements, as recommended by the AUA guidelines. However, given that we did find positive associations between low T and the outcome of interest, we would expect that using a more stringent definition of low T would only result in an even greater effect size. Third, variations in the documentation and coding of diagnoses could contribute to small inconsistencies in HFRS due to its reliance on documented ICD-10 codes. It is plausible that routine diagnosis and documentation of conditions such as delirium may vary between physicians, resulting in variability in patients’ HFRS. Lastly, we did not control for whether patients were taking any T supplementation at the time of surgery, nor did we control for other medications at the time of surgery, such as 5-alpha reductase inhibitors, antiplatelet agents, or anticoagulants. As our study included all patients who had their T measured within a year prior to surgery, it is possible that a subset of patients diagnosed with low T could have been receiving treatment and that an updated follow-up T level after initiating therapy was not captured in our data set. In addition, given the rise of direct-to-consumer T therapy platforms [30], it is becoming increasingly difficult to track whether patients are receiving T therapy outside of their regular medical care. Further prospective studies are needed to confirm these results and determine the utility of T screening and/or supplementation in the preoperative setting.

## Conclusions

Almost half of men undergoing transurethral surgery in our cohort had low T. While low T was not independently associated with frailty, low T was independently associated with a higher risk of 180-day readmission. Therefore, low T may be an independent risk factor for adverse perioperative outcomes after transurethral surgery, indicating a potential role for T screening and treatment in the preoperative setting.
